# Diagnostic value of a point of care bacterial fluorescence imaging device for detecting wound infections in dogs and cats

**DOI:** 10.1111/vsu.14266

**Published:** 2025-05-15

**Authors:** Joanna McCagherty, Jolinda Pollock, Tom W. Maddox, Gavin K. Paterson, Jon L. Hall

**Affiliations:** ^1^ University of Glasgow Small Animal Hospital Glasgow UK; ^2^ Scotland's Rural College Veterinary Services Midlothian UK; ^3^ University of Liverpool Small Animal Teaching Hospital Wirral UK; ^4^ Royal Dick School of Veterinary Studies Division of Veterinary Clinical Sciences Midlothian UK; ^5^ Wear Referrals, Part of Linnaeus Veterinary Ltd Bradbury UK

## Abstract

**Objective:**

To determine if a hand‐held point of care imaging device would improve the detection of bacteria on the surface of wounds in dogs and cats in postoperative and traumatic wounds.

**Study design:**

Clinical prospective study.

**Sample population:**

Three cats and 15 dogs.

**Methods:**

Wounds were swabbed without and with the point of care wound imaging device (WID). Quantitative bacterial culture (QBC), polymerase chain reaction (PCR) and microbiome analysis were performed to assess for any significant difference in the findings of image‐guided and non‐guided sampling methods.

**Results:**

A total of four feline and 17 canine wounds were evaluated. Bacterial fluorescence was detected in all wounds using the point of care WID. Bacterial infections of wounds in dogs and cats was detected by the fluorescent imaging device. No significant difference was identified between the results of QBC and PCR for image‐guided and non‐guided wound swabs (*p* > .05).

**Conclusion:**

The WID was able to detect bacteria on the surface of wounds in dogs and cats, accurately confirming the presence of a clinically relevant wound infection at the time of wound evaluation in all wounds but there was no significant difference in the bacterial yield with guided and non‐guided swabs.

**Clinical significance:**

The WID device was able to confirm the presence of a clinically relevant wound infection in real‐time, enabling the clinician to initiate appropriate systemic and/or topical antibacterial treatment immediately. This study provides proof of concept of the point of care WID in dogs and cats upon which further studies can be based.

**Abbreviations**
AMOVA: analysis of molecular variance
AMR: antimicrobial resistance
AST: antimicrobial susceptability testing
CFU: colony forming unit
DNA: deoxyribonucleic acid
ENA: Euopean Nucleotide Archive
NMDS: non‐metric multidimensional scaling
PCR: polymerase chain reaction
QBC: quantitative bacterial culture
qPCR: qualitative polymoerase chain reaction
SSI: surgical site infection
UK: United Kingdom
WID: wound imaging device

## 1 INTRODUCTION

Traumatic wounds are common in cats and dogs; the true prevalence of these is unknown.^1^ These wounds are typically contaminated; appropriate initial wound management can negate the requirement for systemic antibiotic therapy.^2^ Surgical site infection (SSI) has an incidence of 2%–4.8% following clean procedures in cat and dogs.^3^, ^4^, ^5^ These infections are typically polymicrobial^6^, ^7^ and often reflect contamination from commensal skin bacteria or the environment. They are associated with increased patient morbidity, mortality, treatment cost, extended antibiotic therapy, requirement for revision surgery and owner dissatisfaction.^7^, ^8^, ^9^ Irrespective of traumatic or surgical etiology, bacterial culture from the wound with subsequent antimicrobial susceptibility testing (AST) is the gold standard for diagnosis and selection of appropriate therapy.^9^ However, wound AST results can be challenging to interpret and inappropriate sample site selection and/or culture technique may limit their sensitivity and specificity.^6^

Bacterial contamination with ≥10^4^ colony forming units of bacteria per gram (CFU/g) of tissue will impair wound healing.^6^ Quantitative assessment of bacteria (e.g., real‐time polymerase chain reaction [PCR]) may be more accurate in determining bacterial load compared to semi‐quantitative methods (e.g., assessing numbers of colony forming units, or using subjective description as marked, moderate or sparse cultures) but there is uncertainty as to whether this is clinically relevant and should affect treatment.^6^ Inappropriate use of systemic antimicrobials is a major contributing factor in the dissemination and persistence of antimicrobial resistance (AMR).^10^, ^11^ Antimicrobial stewardship measures to reduce AMR include minimizing systemic antimicrobial use, selection of more targeted systemic antimicrobial therapy^10^, ^11^ and preferential use of topical rather than systemic antimicrobial agents.^11^, ^12^ In the treatment of human wounds, there is a cultural shift towards using topical antiseptics rather than antimicrobial agents.^13^ Optimal antimicrobial stewardship requires accurate surveillance data to identify the true prevalence of clinically significant bacterial wound infections, determine clinically significant infections and assess outcomes following topical treatment with and without systemic antimicrobials.^11^, ^12^

The MolecuLight *i:X* wound imaging device (WID: MolecuLight Inc., Canada) is a commercially available point‐of‐care, handheld diagnostic device and its use has previously been reported in several human studies.^14^, ^15^, ^16^, ^17^, ^18^ The device emits low intensity violet light (wavelength 405 nm) that causes fluorescence of porphyrins and pyoverdines (fluorophores), by‐products of bacterial metabolism, within superficial wound tissue (up to depths of 1.5 mm).^19^ When at high loads (>10^4^ CFU/g), the fluorophores emitted by bacteria can be detected.^14^ The device is calibrated to detect clinically significant (≥10^4^ CFU/g) numbers of bacteria,^19^ consistent with moderate to heavy bacterial growth. According to the manufacturer, normal commensal bacterial burdens at considerably lower levels would not be detected by the device. Therefore, the WID has been shown to be highly predictive in diagnosing wounds with clinically relevant bacterial loads affecting humans.^14^, ^20^, ^21^ Bacterial fluorescence is visible on the video screen display incorporated into the device, enabling real‐time imaging of wounds to be performed, with red fluorescence (or cyan for *Pseudomona* spp.) indicating bacterial loads >10^4^ CFU/mL. Bacteria that are known to fluoresce with the use of the WID (e.g., *Pseudomonas aeruginosa*, *Escherichia coli* and *Staphylococcus* spp.) are listed on the manufacturer's website (https://us.moleculight.com/faq/). When visualized with the WID, collagen and fibrin within the patient skin will emit apple green fluorescence, whilst blood or granulation tissue will appear black as they do not emit any signal when visualized under the violet light emitted by the WID.

The WID has been shown to accurately detect clinically significant numbers of common and relevant bacterial species in burn wounds and chronic wounds in people^19^ and has demonstrated serial reduction in bacterial numbers in wounds. In addition to guiding and improving the accuracy of bacterial sampling,^7^ the device has also been used to guide immediate wound debridement and appropriate antimicrobial therapy.^14^, ^19^ Only a single study has been performed in an experimental animal model^22^ and, to the authors' knowledge, the use of this device has not been investigated in a veterinary clinical environment.

The aim of this study was to determine whether the use of a hand‐held, point of care WID improves the detection of bacteria on the surface of a wound and improves the accuracy of wound sampling for bacterial culture in feline and canine patients.

## 2 MATERIALS AND METHODS

**FIGURE 1:**
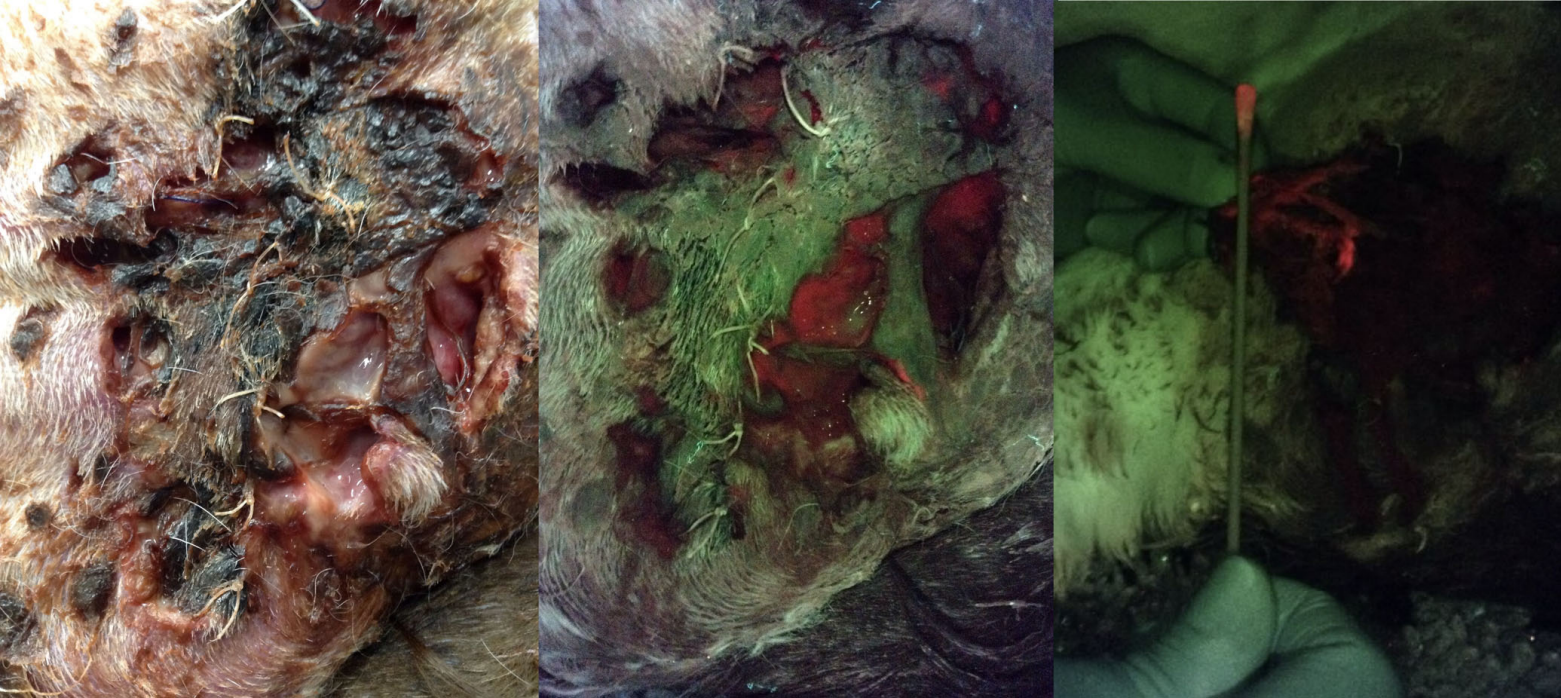
Necrotic flank wound in a dog under different lighting: White light (left) and with violet light using the wound imaging device (WID, center and right). The right image also shows a swab sample being obtained from a region of red fluorescence. Images taken using the WID demonstrate areas of high bacterial burden (indicated by red fluorescence) and host skin and collagen (indicated by apple green fluorescence).

**FIGURE 2:**
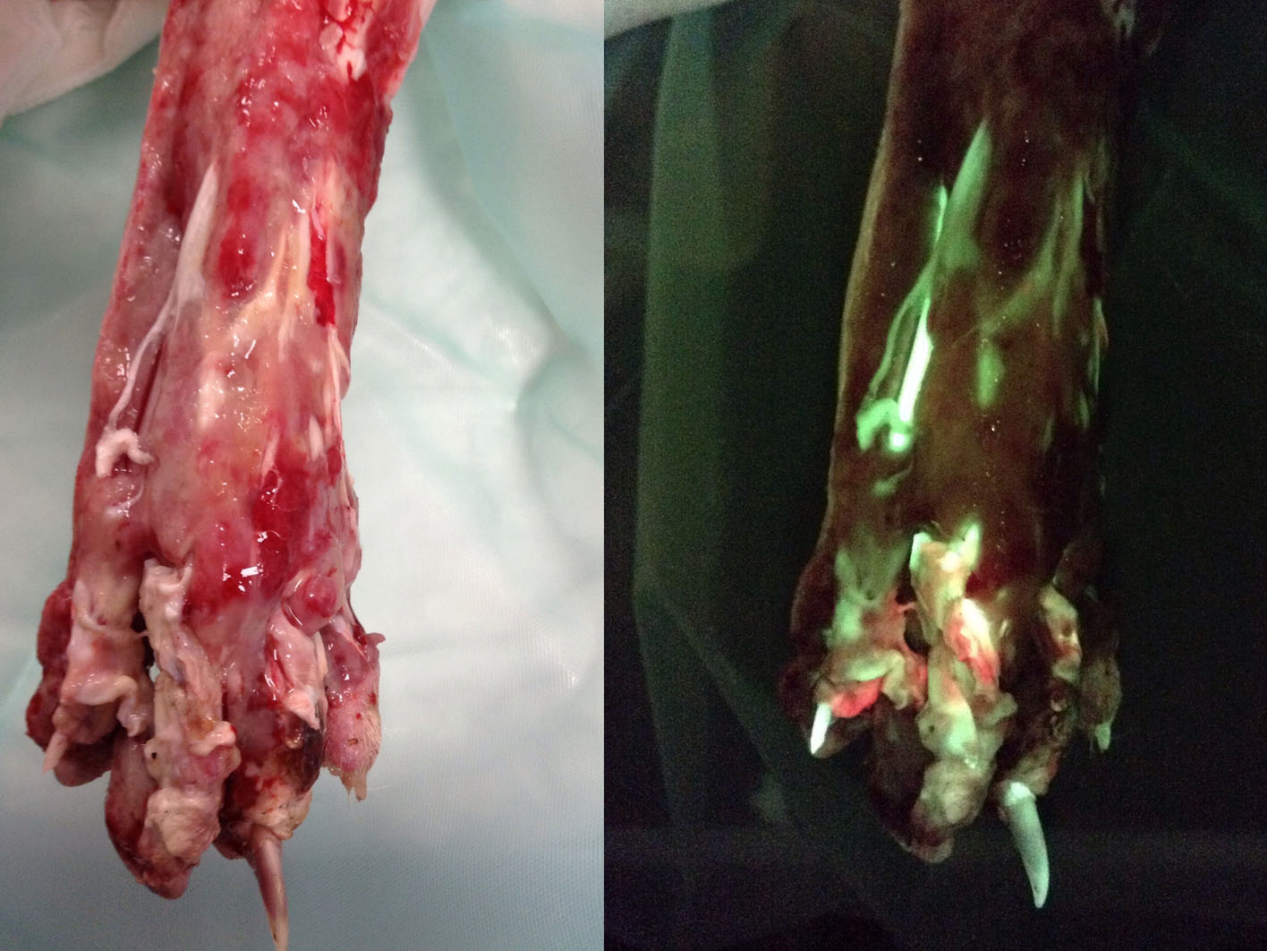
Degloving injury of the pes in a dog under different lighting: White light (left) and with violet light using the wound imaging device (WID) (right). Connective tissues (e.g., tendon) appear as apple green fluorescence. Red fluorescence indicates areas of high bacterial burden.

Ethical approval for the study was granted by the University of Edinburgh Veterinary Ethical Review Committee (approval 40.19). Dogs and cats presenting to the University of Edinburgh Hospital for Small Animals (April to October 2019) and subsequently the University of Glasgow Small Animal Hospital and Wear Referrals (October 2019 to April 2021) with a traumatic wound, incisional dehiscence or surgical site infection were prospectively enrolled in the study. All clients enrolling in the study completed an informed consent form. Data collection was halted between March and October 2020 due to COVID‐19 imposed closure of the microbiology laboratory. The wounds were examined by one of two investigators (JM or JH) in a darkened room using the WID to confirm that at least one portion of the wound fluoresced under violet light as described above. Wound assessment and sampling was performed prior to any intervention such as lavage or debridement. Any amount of detectable fluorescence on the wound surface was considered a positive result and these patients were included in the study. In patients with multiple wounds or wounds where repeat imaging and sampling was performed due to clinical deterioration, the findings of follow‐up analysis of the wound was included in the study. If no fluorescence was observed, the patient was excluded from the study. A photograph of the wound was taken using the camera mode on the WID under violet light in a dark room and another under normal lighting conditions—example images demonstrating wound fluorescence are shown in Figures 1 and 2. A total of four swabs were taken from each wound using the Levine method.^23^ One charcoal (TS/5‐2 swab Amies Charcoal with wood shaft/cotton swab, Technical Service Consultants Limited, Lancashire, UK) and one plain (TS/6‐A260 cotton tip wood shaft swab in tube, Technical Service Consultants Limited) swab were taken from a region of fluorescence (red or cyan colored) on the wound surface using the WID for guidance (guided samples). Subsequently, a colleague, blinded to the result of the wound imaging device assessment, was asked to take one charcoal (Technical Service Consultants Limited) and one plain (Technical Service Consultants Limited) swab from a single region of the wound where they expected the best bacterial yield (non‐guided samples). The charcoal swabs were submitted for microbiology and the plain swabs for PCR analysis. The order in which the charcoal and plain swabs were taken in was not recorded. Details of the case were recorded on a data collection sheet, including: name, case number, signalment, weight, recent medications, cause and age of the wound and whether the wound infection had occurred at the site of a recent surgical intervention.

Data collection sheets were created prior to starting the study. These had randomly assigned labels for the non‐guided and guided samples (A, B, C or D) so that their collection method was not disclosed during laboratory testing. Following sample collection, the swabs were labeled according to the data collection sheet labels.

### 2.1 Quantitative bacterial culture

Charcoal swabs were submitted to the microbiology laboratory at the Royal Dick School of Veterinary Studies, University of Edinburgh for quantitative aerobic and anaerobic bacterial culture. Samples were serial‐diluted and cultured on Columbia horse blood agar and incubated at 37°C for 16 h. Cultures with no growth were incubated for a further 16 h. Identification was performed using VITEK2 (bioMȇrieux, Basingstoke, UK) following the manufacturer's instructions. The results were returned listing the isolated bacterial organism(s) and their burden in colony forming units per milliliter (CFU/mL) along with antimicrobial susceptibility panels where relevant.

### 2.2 DNA extraction

Plain swabs for genomic analysis were frozen at −20°C until sample collection for the study was completed. Samples were thawed at room temperature for 15 min prior to undergoing processing for DNA extraction. DNA extraction was performed using PowerFaecal (QIAGEN, Manchester, UK) DNA isolation kit as per manufacturer's instructions.

### 2.3 16S rRNA gene amplicon sequencing

The extracted DNA samples were then tested to ensure sufficient DNA was present using the Nanodrop 2000 spectrophotometer (Thermo Scientific, UK) and were then stored at −20°C prior to being shipped still frozen to the laboratory (Integrated Microbiome Resource [IMR], Canada) for analysis. The bacterial DNA yield of the PCR swabs was low in all but four of the samples resulting in poor or failed sequencing in the majority of the remaining samples. Some samples required reprocessing to clean the DNA prior to the qPCR being repeated. A clean‐up step was implemented using the Charm Just‐a‐Plate 96 PCR purification and normalization kit (Charm Biotech, San Diego, California). Sequencing of the V6–V8 regions of the 16S rRNA gene was carried out used the sequencing center's standardized procedure (https://github.com/LangilleLab/microbiome_helper/wiki/Microbiome‐Amplicon‐Sequencing‐Workflow).

### 2.4 Statistical analysis

Non‐parametric statistical analysis was performed on the bacterial culture results as the sample numbers were too low to assume normal data distribution. Paired Wilcoxon signed rank tests were performed to compare the total bacterial yield of all species (CFU/mL) from the guided and non‐guided sample from each wound; the bacterial load (CFU/mL) of paired samples where the same bacterial isolate was identified in the guided and non‐guided sample and the number of different bacterial species in the guided and non‐guided samples.

For the 16 s microbiome sequencing results, due to the low bacterial biomass within each swab, sequences were curated carefully during analysis. Sequence data was processed and analyzed using Mothur version 1.48.0,^24^ following the steps in the MiSeq SOP provided on the group's website (https://mothur.org/wiki/miseq_sop/—accessed January 21, 2024). After quality control, five samples were removed from the analysis due to low sequence numbers (i.e., < 296 reads), which had low Good's coverage (i.e., < 90% coverage).

A total of 35 samples and 86 868 reads were taken forward for microbiome analysis. A phylotype‐level analysis was carried out, which generated 515 taxonomic groups. To assess alpha diversity, both Shannon and inverse Simpson diversity indices were calculated, and these data were visualized using the beanplot package^25^ in R (R Core Team, 2022). To assess beta diversity, a distance matrix was compiled using Yue and Clayton theta similarity coefficients^26^ and the statistical significance of groupings assessed by analysis of molecular variance (AMOVA).^27^ Data from the distance matrix were visualized in two dimensions using non‐metric multidimensional scaling (NMDS). To identify taxonomic groups that were significantly different in relative abundance between groupings, Metastats^28^ was used with the *p*‐values being adjusted by Bonferroni correction.

The raw sequence files are available via the European Nucleotide Archive (ENA) under accession number PRJEB73805.

## 3 RESULTS

Overall, samples were obtained from 21 wounds (4 feline and 17 canine). Patient signalment, wound etiology and current medications is presented in Table 1. No wounds were excluded from the study. One cat was presented with two concurrent traumatic wounds and both wounds were included in the study. One dog had the same wound evaluated and sampled 3 days apart due to clinical deterioration of the wound. Another dog had the same wound re‐evaluated around 4 weeks after initial sampling as part of ongoing open wound management. Bacterial culture results and PCR results were not available for 3/21 cases (14.2%) and 1/21, respectively (4.7%). During the data collection period, the authors noticed subjectively that areas of necrotic tissue within the wound tended to be more likely to fluoresce and, in a few cases, the device was reused to guide wound debridement (Table 1).

**TABLE 1:** Information table for animals and wounds, including signalment, wound location and etiology, debridement and medication at the time of sampling.

Wound number	Sample numbers for molecular diagnostics	Bacterial culture available (Y/N)	Species	Breed	Gender	Age (years)	Weight (kg)	Wound location	Wound etiology	Age of wound (if known)	Imaging‐guided wound debridement performed	Current medications at time of sampling
1	1, 2	Y	Feline	Siamese	FN	10	5.1	Body wall	Suspected thermal burn	15d	Y	Clindamicin and meloxicam
2	3, 4	Y	Canine	Bichon Frise	MN	5	12.1	Ventral abdomen	Surgical site infection	6w	N	Maropitant chlorphenamine
3	5, 6	Y	Canine	Cocker Spaniel	MN	5	15.0	Foot	Surgical site infection	2d	N	None
4	7, 8	Y	Canine	Dachshund	M	6	5.0	Upper lip	Surgical site infection	1d	Y	Amoxicillin‐clavulanate
5	9, 10	Y	Canine	Boxer	M	5	32.0	Foot	Surgical site infection	1d	N	Amoxicillin‐clavulanate and chlorphenamine
6	11, 12	Y	Canine	Cocker Spaniel	FN	7	11.9	Distal limb	Suspected snake bite	6d	N	Amoxicillin‐clavulanate and meloxicam
7	13, 14	Y	Canine	Boxer	M	5	32.0	Foot	Surgical site infection	4d	N	Amoxicillin‐clavulanate and chlorphenamine
8	15, 16	Y	Canine	Border Collie	Male	6	27.0	Elbow	Olecranon pressure necrosis	35d	Y	Amoxicillin‐clavulanate
9	N/A	Y	Canine	Pug	FN	4	7.4	Tongue	Necrosis of unknown etiology	5d	N	Amoxicillin‐clavulanate, omeprazole, paracetamol and maropitant
10	A, B	N	Canine					Thoracic wall	Surgical site infection			
11	C, D	N	Canine	French bulldog	FN	10	7.8	Lip	Ulcerated mass	7d	Y	None
12	E, F	Y	Feline	Maine Coon	MN	1	7.6	Tarsus (lateral wound)	Traumatic	7d	N	Cefuroxime and buprenorphine
13	G, H	Y	Feline	Maine Coon	MN	1	7.6	Tarsus (medial wound)	Traumatic	7d	N	Cefuroxime and buprenorphine
14	J, K	Y	Feline	Domestic shorthair	MN	2	3	Medial thigh	Unknown	1d	N	Cefovexin
15	L, M	Y	Canine	Staffordshire Bull Terrier	M	6	19.5	Elbow	Surgical site infection	5d	N	Meloxicam and trazodone
16	N, O	Y	Canine	Dalmatian	FN	8	26.9	Tarsus	Bandage rub	3d	N	Amoxicillin‐clavulanate, meloxicam and tramadol
17	P, Q	Y	Canine	German Shorthaired Pointer	MN	5	26	Body wall	Surgical site infection	6d	Y	Amoxicillin clavulanate, carprofen and metronidazole
18	R, S	Y	Canine	Border Collie	M	6	27.0	Elbow	Olecranon pressure necrosis	7d	N	Amoxicillin‐clavulanate
19	T, U	N	Canine	Rottweiler	M	2	39		Surgical site infection	10d	N	Cefalexin
20	V, W	Y	Canine		M	1	22		Dog bite	3d	N	Amoxicillin‐ clavulanate and meloxicam
21	X, Y	Y	Canine	Dachshund	M	4	8.3	Body wall	Thermal burn	10d	N	Amoxicillin‐clavulanate

*Note*: Patient signalment, medications and wound details.

Abbreviation: d, day.

### 3.1 Bacterial culture

Guided samples yielded zero (*n* = 1), one (*n* = 3), two (*n* = 7), three (*n* = 4), four (*n* = 1) and five (*n* = 2) bacterial species on bacterial culture, whilst non‐guided samples yielded one (*n* = 2), two (*n* = 9), three (*n* = 4), four (*n* = 2) and six (*n* = 1). Guided bacterial culture samples from three cases (16.7%) identified more bacterial species than non‐guided samples, whilst non‐guided samples from seven cases (38.9%) identified more bacterial species than guided samples. The same number of species were identified in both the guided and non‐guided samples in eight (44.4%) cases. There was no significant difference in the number of different bacterial species isolated from guided and non‐guided samples (*p* = .49). When the total bacterial yields (CFU/mL) from the guided and non‐guided samples for each wound were compared, no significant difference was identified (*p* = .47).

There were 14/18 (78%) paired culture samples that had one or more of the same species isolated in the guided and non‐guided samples. This accounted for 25 occurrences of the same bacterial species being present in the guided and non‐guided samples on the same occasion. In these cases, higher burdens of the bacterial isolate (CFU/mL) were reported in the guided sample on 10 occasions and on eight occasions in the non‐guided sample. No significant difference was identified in the bacterial yield (CFU/mL) between paired guided and non‐guided samples (*p* = .83).

### 3.2 Microbiome analysis

**FIGURE 3:**
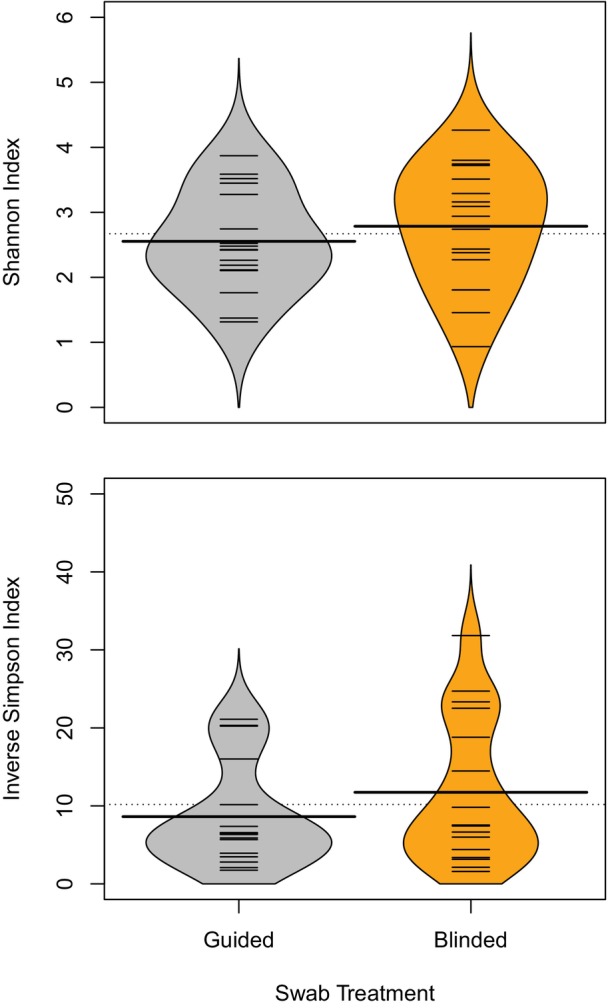
Beanplots visualizing Shannon and inverse Simpson indices from samples in the guided and blinded groups.

Beanplots visualizing the Shannon and inverse Simpson indices are shown in Figure 3. The mean alpha diversity (by both indices) was lower in the guided group, but this difference was not statistically significant for the Shannon index (*p* = .38) or inverse Simpson index (*p* = .30).

**FIGURE 4:**
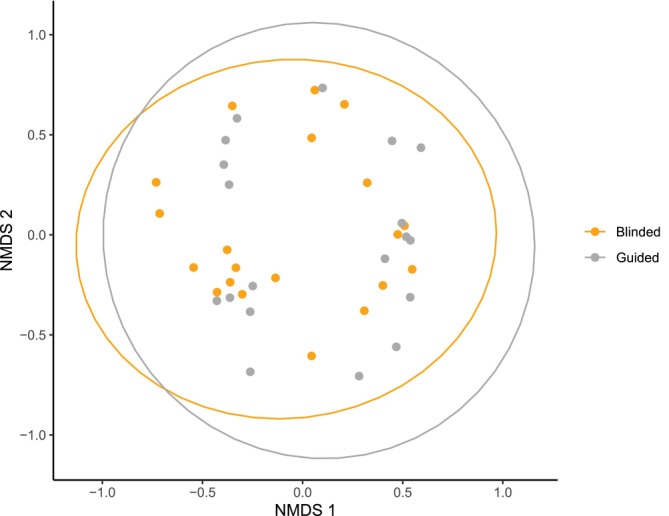
Non‐metric multidimensional scaling (NMDS) plot highlighting bacterial communities within the guided and non‐guided swab types.

The data contained within the distance matrix was visualized using NMDS (Figure 4). There was no evidence of clustering by swab type, which was confirmed statistically by analysis of molecular variance (AMOVA) using the distance matrix as input (*p* = .62). When comparing the non‐guided and guided swabs, there were also no statistically significant differences in relative abundance of any taxonomic groups (Metastats: *p* > .05).

**FIGURE 5:**
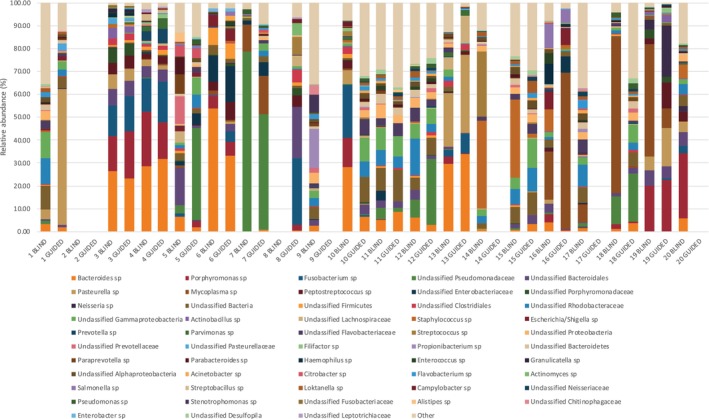
Bar plots demonstrating the relative phylotype abundance for each sample. Adjacent samples (e.g., sample 1 and 2) are paired samples from the same patient representing either guided or non‐guided sampling.

Examining the taxonomic data, 515 phylotypes were assigned in this analysis with nine of these phylotypes representing over 2% of the total number of sequences (Table 2). The relative phylotype abundances for each sample are shown in Figure 5.

**TABLE 2:** The nine phlyotypes representing more than 2% of the total number of taxonomic sequences.

Phylotype	Percentage of total sequences
*Bacteroides* species	17.56%
*Porphyromonas* species	11.98%
*Fusobacterium* species	8.52%
Unclassified Pseudomonadaceae	5.42%
Unclassified Bacteroidales	5.21%
*Pasteurella* species	5.08%
*Mycoplasma* species	4.94%
*Peptostreptococcus* species	3.83%
Unclassified Enterobacteriaceae	2.16%

## 4 DISCUSSION

This study demonstrates that the MolecuLight *i:X* WID is able to detect bacteria on the surface of wounds in dogs and cats. No significant difference was demonstrated in the bacterial yield and burden from swabs obtained under WID guidance compared with non‐guided samples. The majority of the wound infections were polymicrobial and anaerobic bacteria predominated in the bacterial culture and qPCR results.

Bacteria on the superficial surface of canine and feline wounds could be visualized by the WID. In human patients, the WID has been shown to reliably detect bacterial burdens of >10^4^ CFU/mL on the surface of wounds. With the ability to confirm a clinically relevant wound infection using an imaging device, there is evidence to begin empirically selected antimicrobial drugs whilst culture results are pending, avoiding a delay in treatment. Refined selection of the chosen antimicrobial may be aided by topical wound cytology, including gram stain. Early identification of a clinically relevant bacterial infection could also justify the application of topical bactericidal agents, including antiseptics, and justify clinical interventions such as debridement.

We did not demonstrate any significant difference in the bacterial DNA yield or bacterial culture results between WID‐guided samples compared to those collected without image guidance. In human studies, the WID has been shown to target sampling of regions of wounds with higher bacterial burdens, however these studies involve much larger patient numbers.^14^, ^20^, ^21^ In the current study, data was available from 21 infected wounds; this could potentially have introduced a type II error as a result of low statistical power. Additionally, it may be that the wounds affecting cats and dogs are not directly comparable with human patients, in whom more chronic wounds are often described associated with comorbidities (e.g., decubital ulcers in diabetic patients) rather than acute trauma or postoperative complications more commonly presenting in animals and the most common types of wound infections in the current study population. The patients included in the study were only those where there was clinical suspicion that a wound infection was present, and fluorescence was noted using the WID rather than routinely imaging all wounds during the study period because the aim of our investigation was to compare conventional non‐guided wound sampling to image‐guided sampling. This could have resulted in selection bias of heavily contaminated or highly infected wounds where similar results were obtained from both guided and non‐guided samples. Similarly, the clinician taking the sample may have an increased index of suspicion that a particular area of a wound might yield more bacteria without the benefit of fluorescent guidance and in fact, even sample the same area. Whilst this might mean that superiority of bacterial detection using the device is obscured, it does more accurately reflect the relative benefit, or lack of, in taking a sample for culture in clinical veterinary practice versus a highly controlled model. By not including wounds without fluorescence, the sensitivity or specificity of the device for bacterial culture and bacterial yield were not investigated, but this was not the aim of our investigation. The WID has been shown to reliably detect bacterial burdens of >10^4^ CFU/mL on the surface of wounds in people; we determined that sampling wounds without fluorescence, particularly acute traumatic wounds that may yield bacterial growth of commensal or contaminant organisms rather than an organism that may be later the cause of infection,^2^ would interfere with the aim of our study.

The swabs taken for PCR analysis typically had a low bacterial biomass. Good quality samples should contain a minimum of 1 ng/μL of DNA (total). Although all samples were individually assessed for their total DNA content prior to submission and samples contained above 1 ng/μL, this represented both DNA from the wound bacteria and the patient. This resulted in almost all the samples being of poorer quality than expected and additional processing to “clean” the bacterial DNA was required to obtain meaningful results from the majority of the samples tested. A high level of inhibition was detected in most of the samples which was suspected to be due to the patient DNA within the samples. This finding is a limitation of PCR swab testing wounds in veterinary and human patients, particularly where lower bacterial burdens are present, as the patient DNA may interfere with the analysis resulting in a false‐negative result.^29^, ^30^ Bacterial culture is preferred for routine wound swab analysis in both human and veterinary patients^31^; however, in humans, PCR analysis is considered of particular use in chronic wounds where bacteria present in low numbers can contribute to persistent infection. Molecular diagnostics are also of benefit where the bacteria present in wounds is difficult to culture.^31^

The majority of wound infections in this study were found to be polymicrobial. This is consistent with the typical etiology of wound infections occurring as a result of contamination from skin commensals or environmental bacteria.^32^ There were more species of bacteria cultured in the non‐guided sample group which may represent increased detection of contaminant species rather than more accurately detect heavier growth of clinically relevant organisms causing the infection. Anaerobic bacteria were found to predominate in the bacterial populations isolated from the wound swabs, particularly in the PCR analysis, which indicates the importance of performing both aerobic and anaerobic incubation of routine bacterial culture samples. In human patients with chronic wounds, assessment of the wound microbiome is often performed as bacteria in a polymicrobial environment can behave differently compared to a monomicrobial one.^31^

This study had several limitations. First, the authors changed institutions during the sample collection phase of the study, and this meant that samples had to be posted to the laboratory for analysis (bacterial culture) and storage (for PCR samples until sample collection was completed). This resulted in some samples being lost in transit. The study was impacted by COVID‐19 in that caseload at both hospitals was reduced and so fewer cases were available for inclusion. Additionally, the microbiology laboratory at the Royal Dick School of Veterinary Studies was closed for a five‐month period due to COVID‐19 restrictions and data collection was paused. The order of wound sampling was not randomized between cases in that the guided samples were always taken prior to the non‐guided samples. Whilst this did not appear to have any obvious effect on the results, this may have introduced a sampling bias. The wounds were imaged and sampled prior to performing any interventions and may have resulted in surface contaminants or bacteria adherent to necrotic tissue bacteria being sampled. Whilst this is not necessarily representative of the infection present after debridement, the purpose of this study was to assess the ability of the WID to detect bacteria on the surface of wounds in dogs and cats. The wound sampling method used in this study was swabbing. Additional methods such as tissue biopsy for bacterial culture were considered; however, this would have necessitated either heavy sedation or general anesthesia which, in some patients, wound not have been required for the purpose of wound management. Additionally, this would have increased the patient morbidity which could not have been ethically justified purely for the purposes of the study.

Whilst using the device, the authors noted that the presence of fluorescence was more common within necrotic tissue and at, or beneath, the epithelial margins of wounds, which then helped target physical debridement and assessment of completeness of debridement in some cases. Further research would be required to determine the sensitivity, specificity and positive predictive value of the device to detect infection for all wounds, and whether there were benefit to using the device in performing debridement and monitoring response to treatment. The WID has additional uses described in human patients and is considered particularly valuable at guiding wound debridement and ongoing wound management.^21^ To the authors' knowledge, these are yet to be reported in small animal patients. The initial financial outlay for the device (around £7500) may also limit its clinical application in veterinary patients.

Our findings show that the WID can be used to visualize clinically relevant numbers of bacteria on the superficial surface of wounds in dogs and cats. Although our findings did not demonstrate significantly different culture or PCR results compared with non‐guided samples in this study population, it provides proof of concept for use of the device in small animal patients. The WID provides a novel, patient‐side method of wound imaging which enables the clinician to confirm clinically relevant wound infections in dogs and cats. Its use in real‐time can support the use of empirical antibiotic therapy of a confirmed wound infection whilst bacterial culture results are pending, avoiding potential treatment delay and associated patient morbidity.

## AUTHOR CONTRIBUTIONS

McCagherty J, BVMS, DECVS and Hall J, MA, VetMB, DECVS: Designed the study, applied for grant funding, enrolled patients in the study and collected samples. McCagherty J, BVMS, DECVS: Wrote the manuscript in consultation with Hall J, MA, VetMB, DECVS, Maddox TW, BVSc, PhD, DECVDI, Paterson GK, BSc, PhD and Pollock J. McCagherty J, BVMS, DECVS: Performed DNA extractions from the swab samples. Paterson GK, BSc, PhD: Performed bacterial cultures on the swab samples. Pollock J: Evaluated and performed statistical analysis of the genomics data. Maddox TW, BVSc, PhD, DECVDI: Evaluated and performed statistical analysis of the bacterial culture data. McCagherty J, BVMS, DECVS, Hall J, MA, VetMB, DECVS, Paterson GK, BSc, PhD, Maddox TW, BVSc, PhD, DECVDI and Pollock J: Critically reviewed the manuscript during the drafting phases and approved the final manuscript.

## CONFLICT OF INTEREST STATEMENT

The authors declare no conflict of interest.
